# LBoost: A Boosting Algorithm with Application for Epistasis Discovery

**DOI:** 10.1371/journal.pone.0047281

**Published:** 2012-11-08

**Authors:** Bethany J. Wolf, Elizabeth G. Hill, Elizabeth H. Slate, Carola A. Neumann, Emily Kistner-Griffin

**Affiliations:** 1 Division of Biostatistics and Epidemiology, Medical University of South Carolina, Charleston, South Carolina, United States of America; 2 Department of Statistics, Florida State University, Tallahassee, Florida, United States of America; 3 Department of Cell and Molecular Pharmacology, Medical University of South Carolina, Charleston, South Carolina, United States of America; Université de Nantes, France

## Abstract

Many human diseases are attributable to complex interactions among genetic and environmental factors. Statistical tools capable of modeling such complex interactions are necessary to improve identification of genetic factors that increase a patient's risk of disease. Logic Forest (LF), a bagging ensemble algorithm based on logic regression (LR), is able to discover interactions among binary variables predictive of response such as the biologic interactions that predispose individuals to disease. However, LF's ability to recover interactions degrades for more infrequently occurring interactions. A rare genetic interaction may occur if, for example, the interaction increases disease risk in a patient subpopulation that represents only a small proportion of the overall patient population. We present an alternative ensemble adaptation of LR based on boosting rather than bagging called LBoost. We compare the ability of LBoost and LF to identify variable interactions in simulation studies. Results indicate that LBoost is superior to LF for identifying genetic interactions associated with disease that are infrequent in the population. We apply LBoost to a subset of single nucleotide polymorphisms on the PRDX genes from the Cancer Genetic Markers of Susceptibility Breast Cancer Scan to investigate genetic risk for breast cancer. LBoost is publicly available on CRAN as part of the LogicForest package, http://cran.r-project.org/.

## Introduction

Many common diseases are heterogeneous, developing as a result of complex gene-gene and gene-environment interactions [1]–[3]. The heterogeneity of cancer, for example, is well documented and many authors note that distinct genetic patterns in cancer result in significant differences in disease outcome [4]–[6]. While a particular disease pathway may account for a majority of cases, there may be alternative pathways that account for only a small proportion of cases. Statistical methods capable of identifying key components in multiple disease pathways can aid in understanding an individual's risk of developing disease, in disease prognosis, and in prediction of response to therapy [7], [8].

Logic regression (LR) is a single tree-based method capable of modeling high-order interactions [9]. LR generates classification rules by constructing Boolean (and = 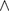, or = 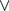, and not = !) combinations of binary (0/1) predictors for classification of a binary response. For example, LR might produce the tree, 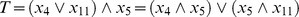, which predicts a response value of 1 if either 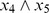 or 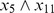 are true. Otherwise, the predicted response is 0. All LR trees can be expressed as a disjunction of conjunctions as in the second expression for tree 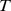. The conjunctive interactions described by the tree are referred to as *prime implicants* (PIs). Tree 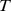 is composed of the two PIs, 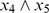 and 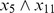, both of size 2 as each includes two variables. LR can identify PIs ranging in size from 1 to 8 predictors, and thus PI is a general term describing main effects and interactions. LR has been used in the development of screening and diagnostic tools for prostate and colorectal cancer, and to identify single nucleotide polymorphisms (SNPs) that confer risk in cardiovascular disease [10]–[13].

Tree-based classifiers are unbiased base classifiers but they are highly variable. The predictive accuracy of a tree-based classifier can be improved by using an ensemble of learners when predicting an observation's class [14]–[16]. The ensemble allows averaging across base learners resulting in an unbiased aggregated learner with reduced variability. One powerful approach to constructing ensemble-based learners is bagging, that is, the construction of classifiers from multiple bootstrap samples drawn from training data. Logic Forest (LF) is a bagged version of LR that generates an ensemble of logic regression-grown trees of varying sizes [17]. LF shows improved predictive performance over LR and is better able to discover PIs significantly associated with response, even in data with predictors measured with error and in data in which not all variables significantly associated with the response are observed. However, the ability of both LR and LF to recover PIs associated with response degrades for infrequently occurring PIs [17]. A rare PI would occur if, for example, the PI is highly predictive of disease for a patient subpopulation that represents only a small proportion of the overall patient population.

Boosting is a powerful alternative algorithm for constructing ensemble learners that reweights the training data at successive iterations to improve prediction of observations poorly classified at previous iterations [18]. In this paper we present a boosted version of LR we refer to as LBoost, and introduce a measure of predictor importance. We compare the performance of LBoost relative to LF considering varying frequency of occurrence for PIs associated with response and varying model complexity. We also apply LBoost to a subset of SNP data from the Cancer Genetic Markers of Susceptibility (CGEMS) Breast Cancer Scan [19]–[21] to investigate genetic risk variants.

## Methods

Define data 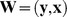 where 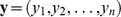 is a vector of 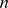 binary responses and 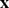 is an 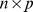 matrix of 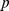 binary predictors with 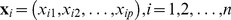. The algorithm for constructing an LBoost model is shown below.

### LBoost Algorithm

For data set 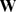

1. Initialize a collection of observation-specific weights 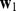 where 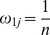 and 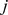 indexes the number of observations, 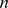.
2. For 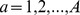 where 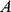 is the number of boosted LR trees constructed from data 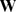

  - Randomly select a positive integer 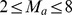 where 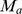 is the maximum number of predictors in an LR tree. (Random selection of tree size has been shown to modestly improve recovery of small PIs [17].)
  - Fit an LR tree, 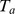, to data 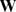 using weights 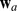 and with no more than 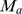 predictors.
  - Compute the weighted error for 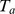 according to:
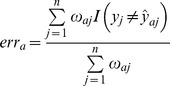
where 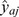 is the predicted value for the 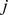
th observation from tree 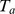

  - Using the weighted error compute a tree-specific weight for tree 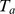 according to:
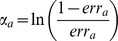

  - Update observation-specific weights according to:
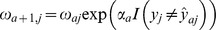

3. The forest of 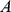 boosted trees is 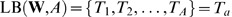.

In step 2b, LBoost fits the LR tree using simulated annealing with misclassification error to choose between LR trees. Simulated annealing is the default search algorithm in LR. Use of misclassification for identifying the “best” LR model limits the number of trees fit at a given iteration of LBoost/LF to one tree with a maximum of 8 predictors.

We also use cross validation (CV) when constructing the forest for development of measures of model fit (Equation 2) and PI importance (Prime Implicant Importance Measures Section). For 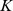-fold CV, let 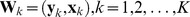 be one of 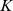 approximately equally sized, non-overlapping subdivisions of the data. Given 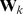, let 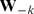 be the collection of all data subdivisions other than 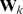 such that 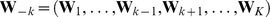, and let 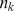 be the number of observations in 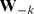. We construct the 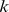
th LBoost model using 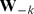 according to the LBoost algorithm and use 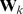 as the 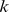
th test data set for the measures of model fit and PI importance. The final LBoost model therefore includes 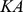 boosted trees and is denoted 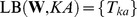.

Now consider an observation 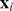 from the 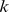
th test data set 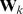. All trees within the boosted forest 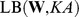 predict class membership for this observation. If predictor values in 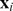 produce a value of 1 for one or more of the PIs in tree 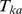 within 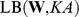, that tree predicts class membership 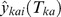 of 1; otherwise the tree predicts the class to be 0.

If we consider test data 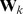 as new data, we can make a CV prediction for the observations in 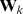 by taking a weighted average of the predictions for those trees in 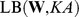 which were constructed from the corresponding training data 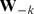. We can use the test data set predictions to calculate an unbiased estimate of model error rate. For observation 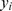 in the test set corresponding to data 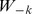 (that is, for 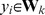), the boosted CV prediction from 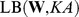 is
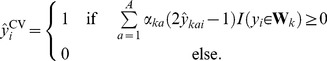
(1)


Since predictions from a logic regression tree take values of either 0 or 1, the expression 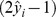 in equation 2 takes on values of 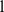 or 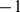, thereby allowing inclusion of all tree-specific weights 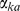 in the final prediction. The CV misclassification rate for 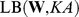 is
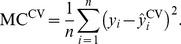
(2)


### Prime Implicant Importance Measure

In contrast to bagging, which applies the base learner to a bootstrap sample of the data, boosting is generally applied to the whole data set making it difficult to define an importance measure. To address this difficulty, we use CV to develop a measure of PI importance that can be estimated from an LBoost model, 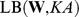. For tree 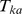, the CV misclassification rate for test data 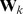 is
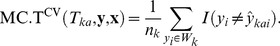
(3)


Let 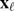 be a PI occurring in tree 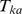, such that 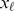 is an 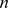-dimensional column vector of 0 s and 1 s corresponding to the PI's value for the 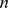 observations. We extract PIs from 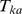 using the *prime.implicant* function available in the logicFS package [22]. Let 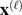 denote the matrix of all PIs in 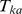 with 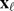 randomly permuted. Let MC.T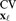 denote the tree-specific misclassification rate for 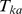 applied to 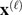. The permutation based variable importance measure for 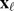 is defined by
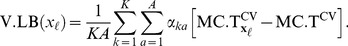
(4)


### Simulation Studies

We conduct several simulations to examine the ability of LF and LBoost to recover PIs representing epistatic interactions between SNPs that are associated with disease. Two types of epistatic interactions are considered for the simulations comparing LBoost and LF (Table 1). An interaction of type 1 confers increased risk of disease when at least one copy of the minor allele is present from both loci; this type 1 interaction is referred to as the jointly dominant-dominant model (DD) [23]–[26]. An interaction of type 2 confers increased risk of disease if two copies of the minor allele are present from both loci; this type 2 interaction is referred to as the jointly recessive-recessive model (RR).

**Table 1:** Two-locus interaction models.

Type 1	AA	Aa	aa	Type 2	AA	Aa	aa
BB	0	0	0	BB	0	0	0
Bb	0	1	1	Bb	0	0	0
bb	0	1	1	bb	0	0	1

Type 1 represents a DD interaction between SNPs a and b while Type 2 represents RR interaction between a and b. A value of 1 indicates SNP combinations conferring increased risk of disease.

We consider three simulation scenarios: (1) the response is associated with a single DD interaction; (2) the response is associated with two DD interactions; and (3) the response is associated with a single RR interaction. We use the liability threshold model [27], [28] to define all interaction models. Specifically, all simulated data are defined by the minor allele frequencies (MAFs) of the risk alleles, the disease prevalence, and the heritability of the epistatic interaction(s). For simplicity, risk alleles in an epistatic interaction have the same MAF. Also, for all simulations, the disease prevalence is set at 0.1 and the heritability for all epistatic interactions is set at 0.02. The disease prevalence was chosen to simulate a common disease such as breast cancer. The population level parameters for specific MAFs, threshold, and heritability are given in Table 2.

**Table 2:** Population values for simulation parameters†.

Model‡	Minor Allele Frequency	Prob(PI+)	Prob(D+ 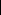 PI+)	Prob(D+ 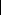 PI−)	OR
	0.1	0.0361	0.2890	0.0930	3.961
Dominant-dominant	0.3	0.2601	0.1460	0.0839	1.866
	0.5	0.5625	0.1213	0.0726	1.763
	0.1	0.0001	1.0000	0.0975	Inf
Recessive-recessive	0.3	0.0081	0.6127	0.0955	14.98
	0.5	0.0625	0.2293	0.0915	2.952

† The disease prevalence is set at 0.1 and heritability is set a 0.02 for all simulations.

‡ MAFs are the same for risk alleles in an epistatic interaction. Prob(PI+) is the probability that a subject has the PI. Prob(D+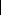
PI+) and Prob(D+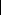
PI−) are the probabilities a subject has disease given that they have the PI and do not have the PI respectively. OR is the population odds ratio given the model, MAF, and heritability.

In addition to the SNPs in the epistatic interaction(s), additional non-causal SNPs are generated such that there are 100 SNPs in the final dataset. Minor allele frequencies for the non-causal SNPs are randomly selected from between 0.05 and 0.5. For simulation scenarios 1 and 2, all SNPs are coded as an indicator for at least one copy of the minor allele. For simulation scenario 3, SNPs are coded as the indicator for two copies of the minor allele. In scenarios 1 and 3 the response is associated with the DD or RR interaction between 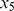 and 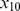, thus the PI of interest is 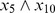. In scenario 2 the response is associated with two independent DD interactions, 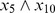 and 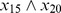.

We consider sample sizes ranging from 400 to 2400, generating 500 datasets for each simulation study. We examine the ability of LF and LBoost to recover the PIs known to be associated with the response using the variable importance measure for LF, V.LF [17], and 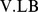 for LBoost. Define 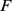 as the set of all PIs identified in either 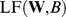 or 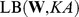. Let 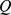 (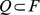) be the set of 20 PIs in 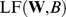 or 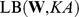 with maximum absolute V.LF and V.LB (4) values, respectively. We say that the PI 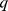, known to be associated with disease, has been recovered when 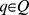. We select the top 20 identified PIs because in the context of studying gene-gene interactions, 20 interactions represents 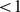
% of all possible 2 locus combinations given 100 geneotyped SNPs.

We use the Logic Forest package in R v.2.14.1 [29] with simulated annealing optimization to fit all LF models [9], [17]. For LBoost we use 5-fold CV and construct 20 trees for each dataset 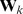 resulting in an LBoost model with 100 LR trees. For comparisons, all LBoost and LF models include the same number of LR trees. The same starting and ending annealing temperatures are selected for LF and LBoost. The starting temperature of 2 is selected such that approximately 90% of “new” models are accepted. The final temperature of 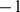 is set to achieve a score where fewer than 5% of new models are accepted. The cooling schedule is set so that 50,000 iterations are required to get from start to end temperaure. Increasing the number of iterations to 250,000 does not affect our findings. With these settings, the LBoost algorithm constructs a model in less than a minute on a Windows 2.26 GHz machine.

## Results

### Scenario 1: One Dominant-Dominant Interaction

**Figure 1:**
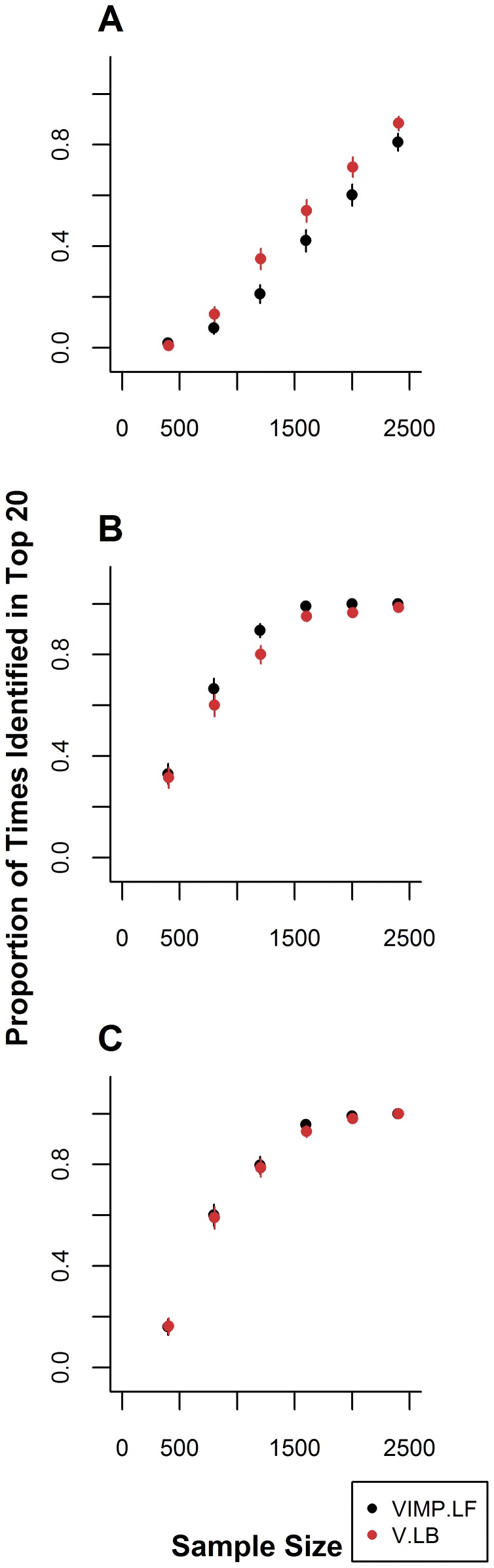
Recovery of the dominant-dominant interaction 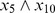 for MAFs of 0.1, 0.3, and 0.5. Each panel shows the proportion of times in 500 simulation runs the DD PI 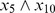 is recovered among the top 20 PIs by each method for different MAFs for 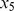 and 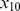. A) MAFs for 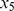 and 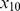 are 0.1, panel B) MAFs for 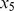 and 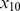 are 0.3, and panel C) MAFs for 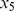 and 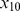 are 0.5. Error bars represent 95% confidence intervals.

n scenario 1, we investigate the ability of LBoost and LF to recover a single DD interaction that is associated with the response from among 100 binary variables. The minor allele frequencies of 0.1, 0.3, and 0.5 are considered. In data in which the MAFs for 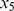 and 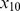 were 0.1, LBoost identified the combination 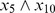 more frequently than LF, although this difference was only significant for 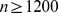 (Figure 1A). LBoost recovers 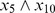 in a maximum of 88.4% of simulations, while LF recovers this PI in a maximum of 81.0% of simulation runs. When the minor allele frequencies for 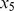 and 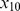 are increased to 0.3, the ability of both LF and LBoost to recover 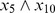 improves. Under these conditions, LF recovers 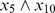 significantly more frequently than LBoost for 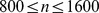 (Figure 1B). Both LF and LBoost recover the PI in 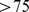
% of simulation runs for 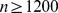 and in more than 90% of simulation runs for 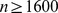. In data in which the MAFs for 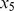 and 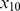 are 0.5, LF and LBoost identify 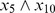 equally well, recovering this PI in 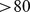
% of simulation runs for 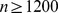.

### Scenario 2: Two Independent Dominant-Dominant Interactions

**Figure 2:**
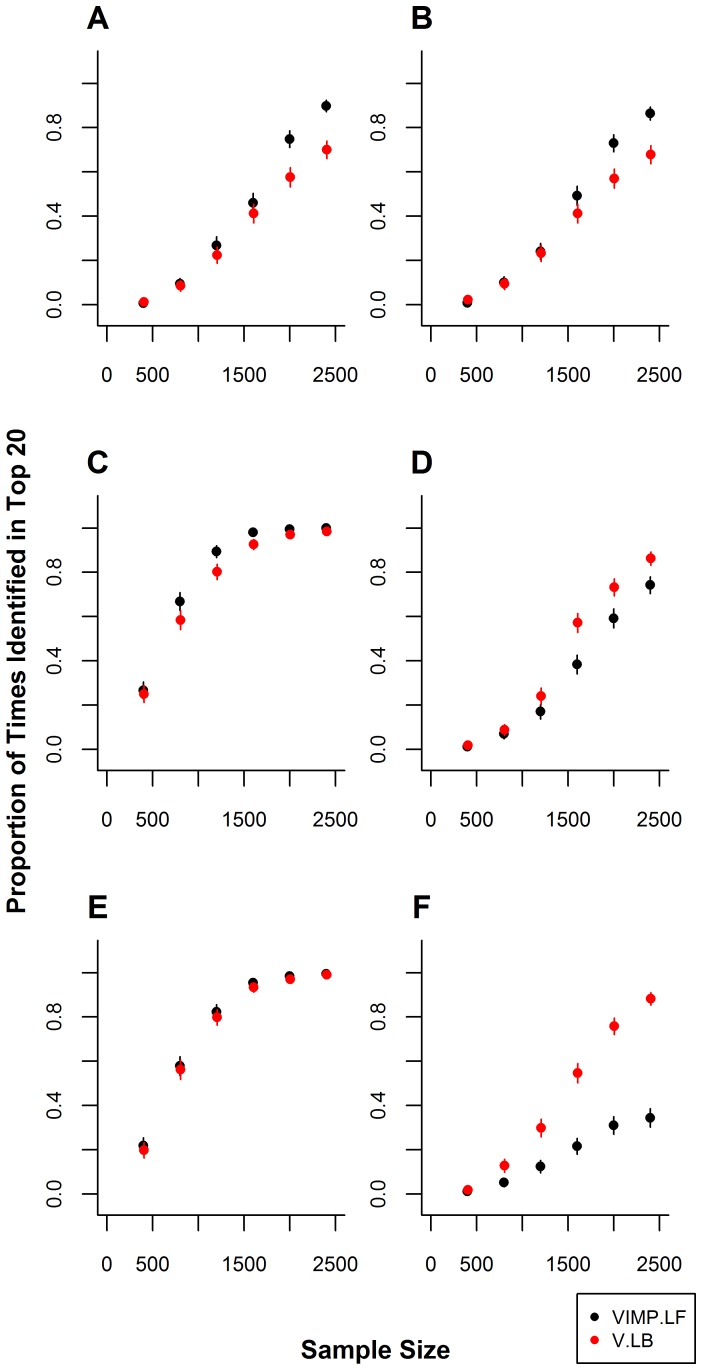
Recovery of the dominant-dominant interactions 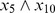 and 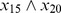 for MAFs of 0.1, 0.3, and 0.5. Each panel shows the proportion of times in 500 simulation runs the DD PIs 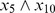 and 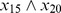 are recovered among the top 20 PIs by each method for different MAFs. Specifically, Panels A) and B) show the proportion of times each method recovers 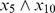 and 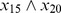 respectively when MAFs for 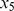 and 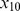 are 0.1 and MAFs for 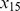 and 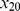 are 0.1. Panels C) and D) show the proportion of times each method recovers 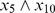 and 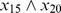 respectively when MAFs for 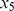 and 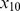 are 0.3 and MAFs for 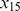 and 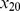 are 0.1. Panels E) and F) show the proportion of times each methods recovers 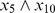 and 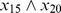 respectively when MAFs for 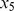 and 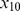 are 0.5 and MAFs for 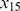 and 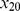 are 0.1. Error bars represent 95% confidence intervals.

In the second scenario, we investigate the ability of LBoost and LF to recover 2 DD interactions that occur with different frequency. The MAFs for the two SNPs in the PI 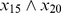 are held constant at 0.1 while the MAFs for 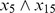 are set at 0.1, 0.3 or 0.5. In the first case, the MAFs for 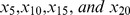 are set at 0.1, thus the expected frequency of occurrence of the two PIs 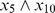 and 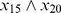 are equivalent. For 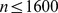, LF and LBoost recover the PIs equally well. However LF recovers both PIs significantly more frequently than LBoost for 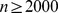 (see Figures 2A and 2B). LBoost recovers both PIs in 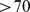
% of simulation runs for 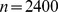, however LF recovers both PIs 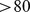
% of simulation runs for the largest sample size.

In the second case, the MAFs for 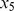 and 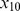 are increased to 0.3, but the frequencies of 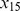 and 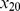 are held at 0.1. In this case the PI 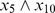 occurs more frequently than 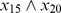. Both LF and LBoost recover 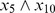 more frequently than in the previous case. However, LF recovers this PI significantly more frequently than LBoost for 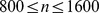 (Figure 2C). Both methods identify this PI in 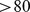
% of simulation runs for 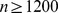. LBoost identifies the less frequently occurring PI, 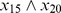, significantly more often than LF for 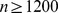 (Figure 2D).

In the third case, the MAFs for 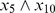 are increased to 0.5 holding the frequencies for 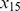 and 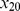 at 0.1. There is no significant difference in the proportion of times LF and LBoost recover 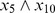. Both methods recover this PI in 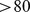
% of simulation runs for 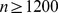 (Figure 2E). However, LBoost recovers 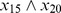 significantly more frequently than LF for 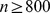 (Figure 2F).

We also compare LBoost models with varying K-fold CV (K = 5, 10, and 20) with forest size KA = 100 for cases 1 and 3 for two independent DD interactions. In case 1 (MAF 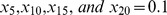), LBoost identifies both PIs significantly more frequently using 5-fold CV relative to 20-fold CV for 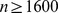 (Figure S1, panels A and B). However, there is not a significant difference between 5 and 10-fold CV. In case 3 ( MAF 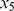 and 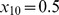 and MAF 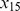 and 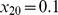), there is not a significant difference in the proportion of times LBoost recovers 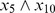 or 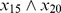 for 5, 10, and 20-fold CV at any sample size (Figure S1, panels C and D).

Additionally we examine the performance of LBoost in models with 100 (with 5-fold CV) and 200 (with 10-fold CV) trees holding the ratio of total number of trees, 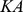, to number of CV data set, 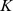, constant at 20∶1. Increasing the number of trees from 100 to 200 improves the proportion of times LBoost recovers the PIs in case 1 for 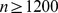 though the difference is not significant (Figure S2, panels A and B). In case 3, there is no significant differences in the proportion of times LBoost recovers 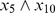 in models with 100 versus 200 trees (Figure S2, Panel C). However, LBoost identifies 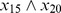 significantly more often in models with 200 trees for 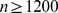 though the difference is only significant for 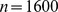.

### Scenario 3: One Recessive-Recessive Interaction

In simulation scenario 3, we consider the ability of LF and LBoost to recover a single RR interaction. The probability of occurrence of the PI given disease status is less than in previous scenarios. As in the first scenario we consider MAFs of 0.1, 0.3, and 0.5 for 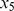 and 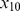. When both 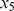 and 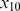 have MAFs of 0.1, the probability of observing 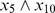 given a subject is disease positive is 0.1%. This PI occurs so infrequently, neither LBoost nor LF identified 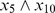 at any sample size under consideration (results not shown).

**Figure 3:**
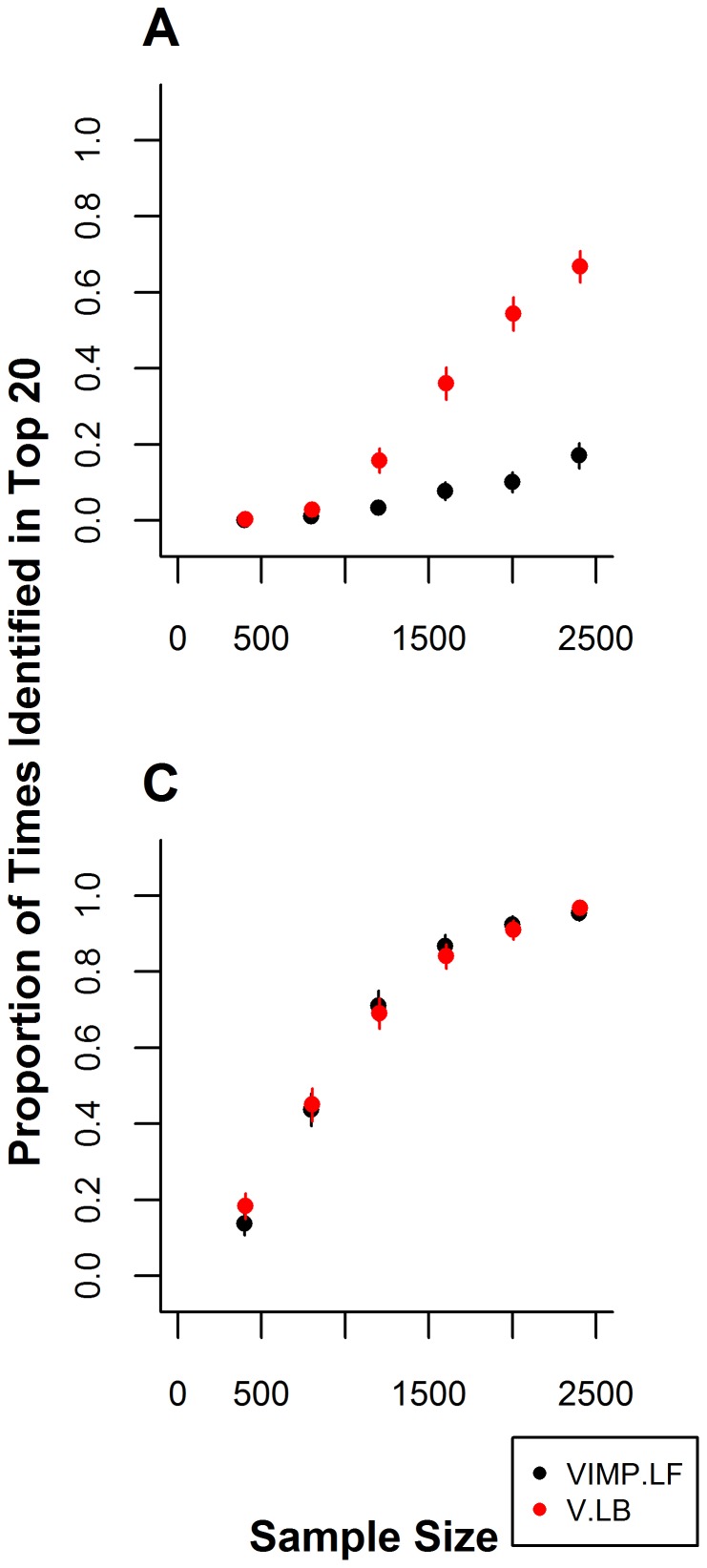
Recovery of the recessive-recessive interaction 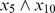 for MAFs 0.3 and 0.5. Each panel shows the proportion of times in 500 simulation runs the RR PI 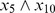 is recovered among the top 20 PIs by each method for different MAFs for 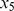 and 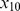. Panel A) MAFs for 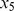 and 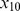 are 0.3 and panel B) MAFs for 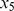 and 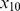 are 0.5. Error bars represent 95% confidence intervals.

When the MAFs of 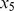 and 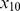 are increased to 0.3, the probability of the PI given a subject has disease increases to approximately 5%. In this case, LBoost identifies 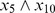 significantly more frequently than LF for 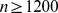 (Figure 3, Panel A). LBoost identified this PI in a maximum of 67% of simulation runs, however LF identified it in a maximum of only 17% of simulation runs. When the minor allele frequencies for 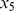 and 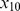 are increased to 0.5, the probability of 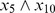 given the subject has disease increases to 0.1433. The ability of both LF and LBoost to identify this PI is improved and both recover this PI in 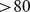
% of simulation runs for 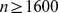 (Figure 3, Panel B). There is not a significant difference in the proportion of times each method recovers this PI at any sample size.

We also examine the performance of LF and LBoost in models with 100 (5-fold CV) and 200 (10-fold CV) trees holding the ratio of total number of trees, 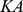, to number of CV data set, 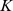, constant at 20∶1. Increasing the number of trees from 100 to 200 significantly improves the proportion of times LBoost recovers the 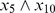 for 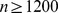 (Figure S3). In models with 200 trees, LBoost recovers 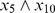 in 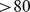
% of simulations for 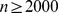 but recovers the PI in a maximum of 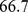
% of simulations when LBoost models include 100 trees. Increasing the number of trees in a LF model does not significantly impact the ability of LF to recover 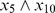 (Figure S3).

### Summary of Simulation Results

LF and LBoost exhibit similar ability to recover frequently occurring PIs. However, LBoost performs better than LF when PIs occur rarely (5 to 10% of the time among individuals with disease) and is better at recovering less frequent PIs in the presence of a frequently occurring PI. There is also a trend towards improved recovery of PIs with increasing the number of trees in an LBoost model regardless of frequency.

### CGEMS Analysis

Peroxiredoxins (Prdxs) are a newly identified group of peroxidases upregulated in breast cancer [30]–[33]. No genetic analysis has been done so far to investigate the genomic integrity of the PRDX genes in breast or any other cancer. We investigate single nucleotide polymorphisms (SNPs) in the Cancer Genetic Markers of Susceptibility study (CGEMS) [19], [20] data, available in dbGaP (dbGaP accession number: ps000147.v1.p1). In total, 94 SNPs that are within 50 kb of the six PRDX genes are included in the LF and LBoost analyses.

The CGEMS study is an NCI-sponsored project begun in 2005 as a pilot study to identify genetic variants associated with increased risk of breast and prostate cancers. The CGEMS breast cancer data was derived from incident post-menopausal breast cancer cases in the Nurses' Health Study (NHS) arising between 1990 and 2004 [21]. Women in the CGEMS study provided a blood sample in 1989 or 1990 as part of the NHS and were cancer free at the time of sampling. In total 1145 incident cases were matched to 1142 controls from the NHS on age, blood collection time, ethnicity (all are self-reported Caucasian), and menopausal status at blood draw (all are menopausal at blood draw). Participants were genotyped using the Ilumina HumanHap550 chip. For each subject approximately 528,000 SNPs were genotyped providing coverage of 90% of the common SNPs.

Our analysis data comprised 94 SNPs on the six PRDX genes, coded for analysis by an indicator variable that takes value 1 if the subject has at least one copy of the minor allele in order to test the dominant effect of the minor allele. The LF and LBoost models constructed for these data both contain 100 trees. The LBoost model uses 5-fold cross-validation in model construction. The LF and LBoost models each identified over 300 unique PIs involving the 94 SNPs. PI importance was ranked from least to greatest according the VIMP.LF for the LF model and according to 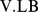 for LBoost. Empirical p-values were obtained for all PIs using a permutation approach.

Both LF and LBoost identified the PIs *rs11198819* (p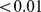) and *rs11198819* 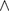 *rs2297696* (p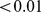) among the top 5 most important PIs. The SNP *rs2297696* is upstream of PRDX3 on the sideroflexin 4 gene. The SNP *rs11198819* is downstream from PRDX3 in a non-coding region however, it is in strong linkage disequilibrium (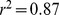) with *rs3749562* which is on the PRDX3 gene. The remaining PIs in the LF model included *rs11198819* in conjunction with at least one additional SNP. LBoost identified two additional PIs not identified by LF, *rs1205171* (p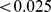) and *rs1205171* 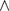 *rs1461024* (p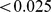). The SNP *rs1205171* is found on the PRDX2 gene and *rs1461024* is found on the PRDX6 gene.

The moderate significance of these SNPs and SNP interactions is likely due to the fact that the PRDX family of genes does not play a dominant role in breast cancer. However, these results suggest possible associations of genetic variants within the PRDX family of genes with breast cancer. Additionally, LBoost identified SNPs not identified by LF. Further laboratory studies are necessary to explore the SNP interactions identified by LF and LBoost.

## Discussion

Logic Forest, an ensemble adaptation of logic regression, has the ability to model complex interactions among binary predictors to describe disease state. However, LF is less adept in recovering rare PIs associated with disease, particularly in the presence of more frequent, predictive PIs. We introduced a boosting adaptation of LR referred to as LBoost in order to address this weakness of LF. Additionally we presented a predictor/PI importance measure based on permutation of a predictor or PI in the data, 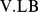.

The results of the simulation study indicate that the ability of LF and LBoost to recover PIs associated with disease depends on the frequency with which a PI occurs in subjects that have disease and whether or not an additional predictive PI is present. In the scenario where the data only included a single DD interaction, LF and LBoost performed similarly, although LBoost showed modest improvement over LF in recovering 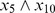 when the minor allele frequency was low (0.1). In this case, the PI occurred in approximately 10% of subjects with disease. However, when the minor allele frequency increased to 0.3 (PI occurring in approximately 38% of subjects with disease) LF has better ability to recover the PI at smaller sample sizes. The greatest difference in ability to recover a single PI occurred in data where the interaction of interest was a recessive-recessive interaction in which the MAFs for 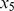 and 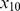 were 0.3. In this case only 5% of subjects with disease were expected to have the PI 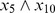 and LBoost identified this PI significantly more often than LF for 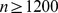.

In data with two interactions, LBoost recovered the less frequently occurring PI, 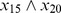 significantly more frequently than LF and performed similarly to LF in recovering the more frequently occurring PI. This difference in the ability to recover the rarer PI is more pronounced as the difference in frequency of occurrence between the two PIs increases.

For a fixed number of trees, increasing the number of CV sets, 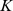, in an LBoost model moderately improves the ability of LBoost to identify frequent PIs. However, increasing the number of CV sets also decreases the ability LBoost to identify rare PIs. This effect is most pronounced in data with two or more PIs where both PIs are infrequent. However, the impact of varying the number of CV sets is small and choice of 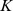 and 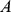 should not greatly impact the ability of LBoost to identify PIs. From experience we have found that selecting the total number of trees, 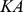, and the number of CV data sets, 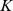, such that the ratio of total trees to number of CV data sets 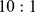 provides good balance for identifying frequent and rare PIs.

Increasing the total number of trees improves LBoost's ability to identify rare PIs. This effect is most noticeable when the PI is rare (i.e. the PI occurs in 5% of the cases), and is not evident for PIs that occur with greater than 10% frequency among cases. However, little additional computational time is necessary when increasing the forest size from 100 to 200 trees and therefore is advisable.

Both LBoost and LF are best suited for targeted investigation of SNP interactions associated with disease (e.g. pathway analysis). LBoost performs similarly to LF for frequently occurring PIs although LF performs better for mid-range sample sizes (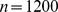 to 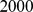). However, LBoost is better able than LF to identify rare interactions that occur in approximately 5–10% of subjects with disease. LBoost is also better adapted to identify multiple PIs in situations where PI frequency varies among the PIs predictive of disease, a scenario more closely resembling a complex disease such as cancer. Since we can not know the data structure *a priori*, it is helpful to explore the predictor space using both methods.

Although we described the LBoost algorithm using LR with misclassification as the measure of goodness of fit, there are additional fit measures available in LR (e.g. deviance and least squares). There are also search algorithms other than simulated annealing that could be used to search for logical combinations of binary predictors. In subsequent work we will explore use of other LR measures of fit and additional search algorithms for identifying combinations of binary predictors in constructing LBoost models.

## Supporting Information

Figure S1
**Recovery of DD interactions** 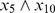 **and** 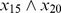 **in LBoost models with 100 trees and 5, 10, or 20-fold CV.** Each panel shows the proportion of times in 500 simulation runs the DD PIs 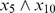 and 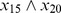 are recovered among the top 20 PIs by LBoost when the number of CV sets, 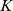, is set to either 5, 10 or 20. The total number of LR trees in all models is held constant at 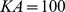. In all panels, black is LBoost with 5-fold CV, red is LBoost with 10-fold CV, and green is LBoost with 20-fold CV. Specifically, Panels A) and B) show the proportion of times LBoost recovers 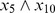 and 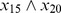 respectively for different values of 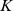 when MAFs for 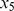 and 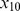 are 0.1 and MAFs for 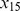 and 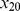 are 0.1. Panels C) and D) show the proportion of times LBoost recovers 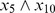 and 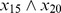 respectively for different values of 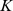 when MAFs for 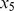 and 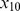 are 0.5 and MAFs for 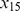 and 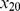 are 0.1. Error bars represent 95% confidence intervals.(BMP)

Figure S2
**Recovery of DD interactions** 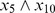 **and** 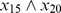 **in LBoost models with 100 or 200 trees.** Each panel shows the proportion of times in 500 simulation runs the DD PIs 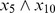 and 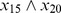 are recovered among the top 20 PIs by LBoost when the number of LR trees in the LBoost model is either 100 or 200. We use 5-fold CV in LBoost models with 100 LR trees and 10-fold CV in models with 200 trees. Thus the ratio of total trees to 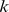-fold CV is held constant at 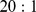. In all panels, black is LBoost with 100 trees and red is LBoost models with 200 trees. Specifically, Panels A) and B) show the proportion of times LBoost recovers 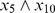 and 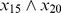 respectively for models with 100 and 200 trees when MAFs for 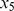 and 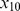 are 0.1 and MAFs for 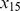 and 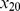 are 0.1. Panels C) and D) show the proportion of times LBoost recovers 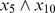 and 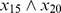 respectively for models with 100 and 200 trees when MAFs for 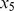 and 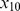 are 0.5 and MAFs for 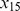 and 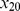 are 0.1. Error bars represent 95% confidence intervals.(BMP)

Figure S3
**Recovery of the RR interaction** 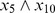 **for MAF of 0.1 in LBoost models with 100 or 200 trees.** The graph shows the proportion of times in 500 simulation runs the RR PI 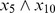 is recovered among the top 20 PIs by both when the number of LR trees in the LBoost or LF models is either 100 or 200. We use 5-fold CV in LBoost models with 100 LR trees and 10-fold CV in models with 200 trees. Thus the ratio of total trees to 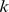-fold CV in all LBoost models is held constant at 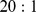. In all panels, black is LF models with 100 trees, red is LF models with 200 trees, green is LBoost models with 100 trees, and blue is LBoost models with 200 trees. Error bars represent 95% confidence intervals.(BMP)
