# Supplementary figures and images for: LBoost: A Boosting Algorithm with Application for Epistasis Discovery
Source: PLoS One. 2012 Nov 8;7(11):e47281. doi: 10.1371/journal.pone.0047281 (PMC3493573; doi:10.1371/journal.pone.0047281)

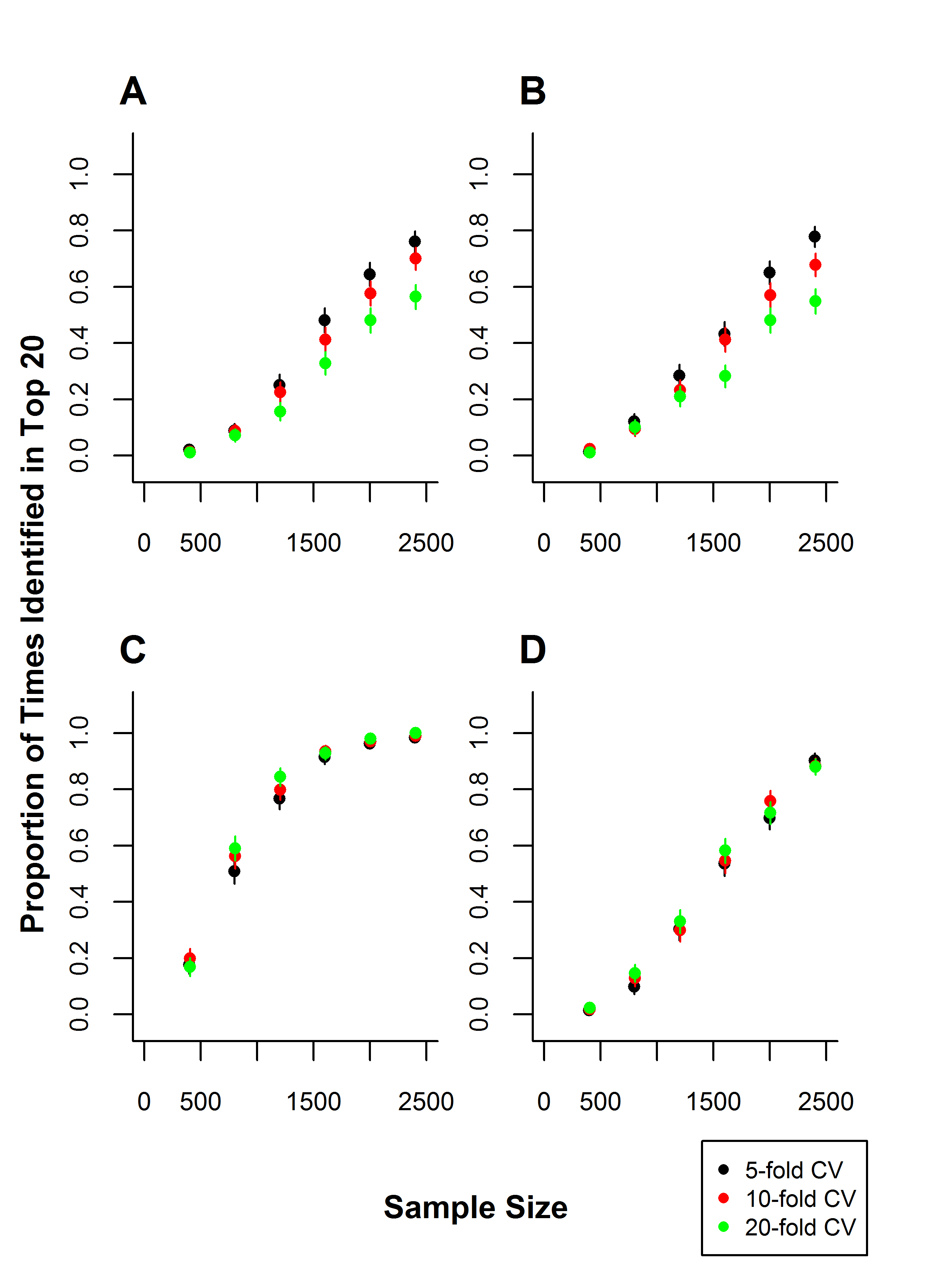

Supplement: Figure S1 — Recovery of DD interactions and in LBoost models with 100 trees and 5, 10, or 20-fold CV. Each panel shows the proportion of times in 500 simulation runs the DD PIs and are recovered among the top 20 PIs by LBoost when the number of CV sets, , is set to either 5, 10 or 20. The total number of LR trees in all models is held constant at . In all panels, black is LBoost with 5-fold CV, red is LBoost with 10-fold CV, and green is LBoost with 20-fold CV. Specifically, Panels A) and B) show the proportion of times LBoost recovers and respectively for different values of when MAFs for and are 0.1 and MAFs for and are 0.1. Panels C) and D) show the proportion of times LBoost recovers and respectively for different values of when MAFs for and are 0.5 and MAFs for and are 0.1. Error bars represent 95% confidence intervals. (BMP) [file pone.0047281.s001.bmp]

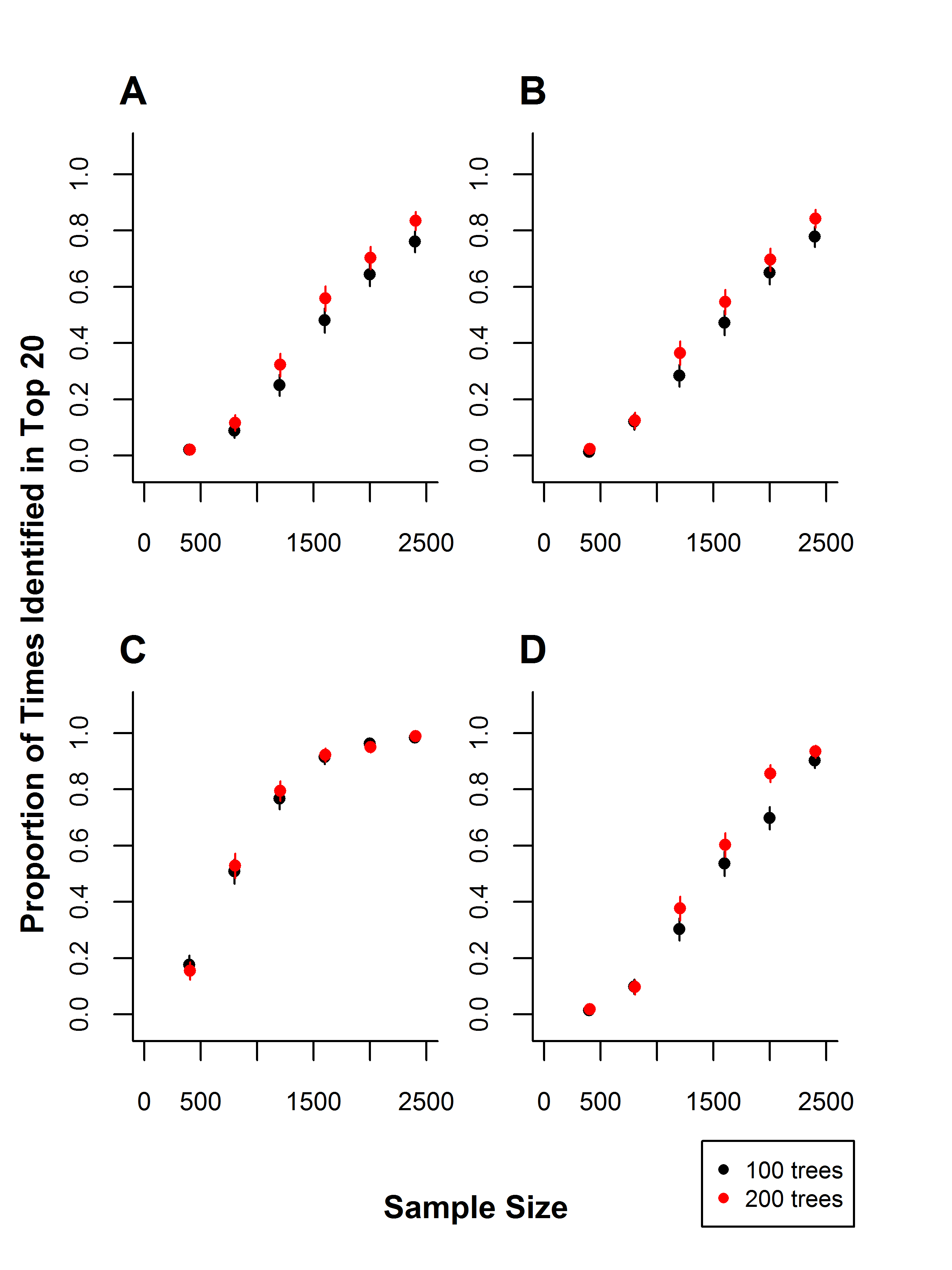

Supplement: Figure S2 — Recovery of DD interactions and in LBoost models with 100 or 200 trees. Each panel shows the proportion of times in 500 simulation runs the DD PIs and are recovered among the top 20 PIs by LBoost when the number of LR trees in the LBoost model is either 100 or 200. We use 5-fold CV in LBoost models with 100 LR trees and 10-fold CV in models with 200 trees. Thus the ratio of total trees to -fold CV is held constant at . In all panels, black is LBoost with 100 trees and red is LBoost models with 200 trees. Specifically, Panels A) and B) show the proportion of times LBoost recovers and respectively for models with 100 and 200 trees when MAFs for and are 0.1 and MAFs for and are 0.1. Panels C) and D) show the proportion of times LBoost recovers and respectively for models with 100 and 200 trees when MAFs for and are 0.5 and MAFs for and are 0.1. Error bars represent 95% confidence intervals. (BMP) [file pone.0047281.s002.bmp]

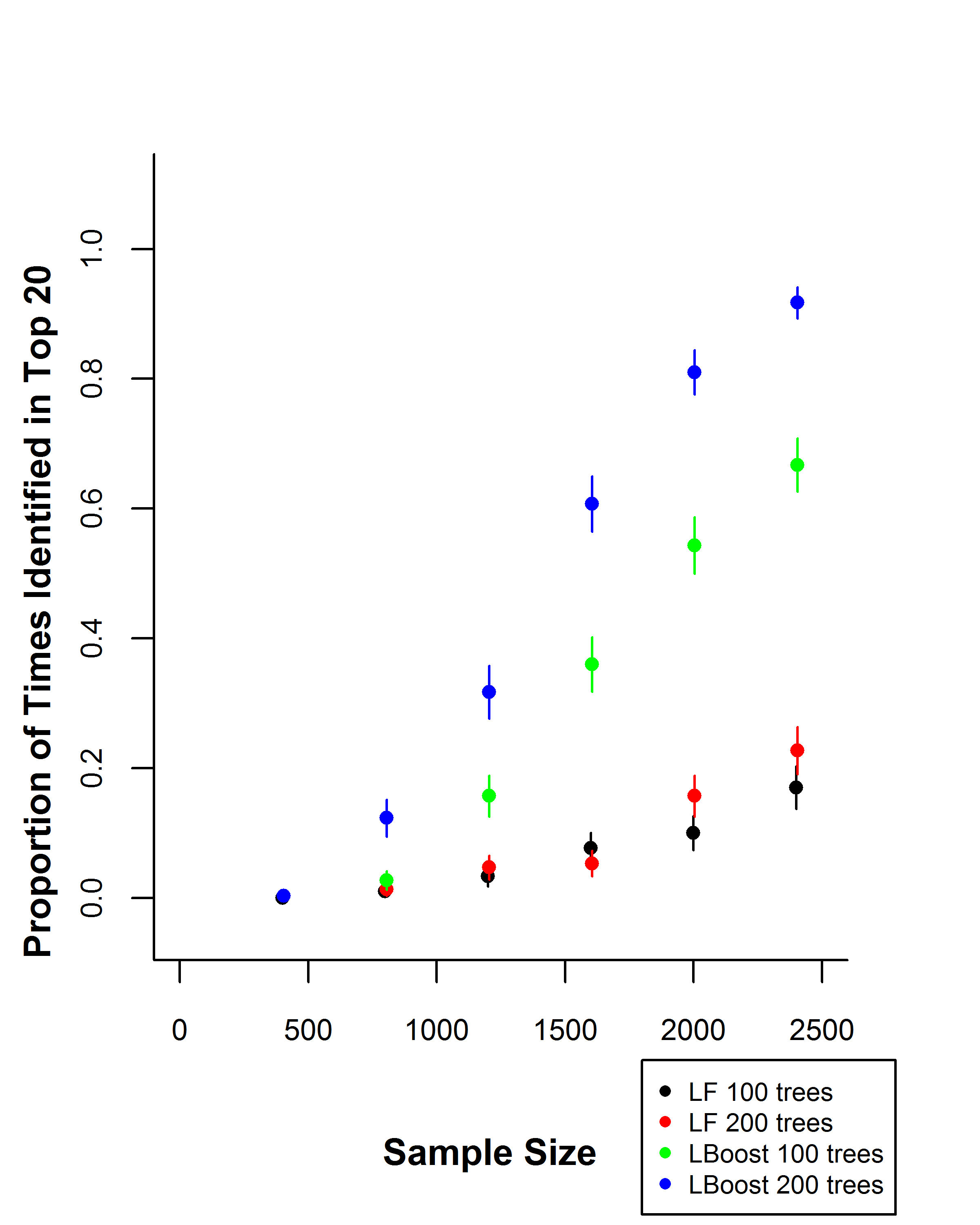

Supplement: Figure S3 — Recovery of the RR interaction for MAF of 0.1 in LBoost models with 100 or 200 trees. The graph shows the proportion of times in 500 simulation runs the RR PI is recovered among the top 20 PIs by both when the number of LR trees in the LBoost or LF models is either 100 or 200. We use 5-fold CV in LBoost models with 100 LR trees and 10-fold CV in models with 200 trees. Thus the ratio of total trees to -fold CV in all LBoost models is held constant at . In all panels, black is LF models with 100 trees, red is LF models with 200 trees, green is LBoost models with 100 trees, and blue is LBoost models with 200 trees. Error bars represent 95% confidence intervals. (BMP) [file pone.0047281.s003.bmp]
